# Machine Learning-Based Gesture Recognition Glove: Design and Implementation

**DOI:** 10.3390/s24186157

**Published:** 2024-09-23

**Authors:** Anna Filipowska, Wojciech Filipowski, Paweł Raif, Marcin Pieniążek, Julia Bodak, Piotr Ferst, Kamil Pilarski, Szymon Sieciński, Rafał Jan Doniec, Julia Mieszczanin, Emilia Skwarek, Katarzyna Bryzik, Maciej Henkel, Marcin Grzegorzek

**Affiliations:** 1 Department of Medical Informatics and Artificial Intelligence, Faculty of Biomedical Engineering, Silesian University of Technology, Roosevelta 40, 41-800 Zabrze, Poland; anna.filipowska@polsl.pl (A.F.); pawel.raif@polsl.pl (P.R.); marcpie568@student.polsl.pl (M.P.); julibod178@student.polsl.pl (J.B.); piotfer581@student.polsl.pl (P.F.); kp272628@student.polsl.pl (K.P.); rafal.doniec@polsl.pl (R.J.D.); julimie273@student.polsl.pl (J.M.); emilskw843@student.polsl.pl (E.S.); katabry222@student.polsl.pl (K.B.); 2 Department of Telecommunications and Teleinformatics, Faculty of Automatic Control, Electronics and Computer Science, Silesian University of Technology, Akademicka 16, 44-100 Gliwice, Poland; wojciech.filipowski@polsl.pl; 3 Łukasiewicz Research Network—Krakow Institute of Technology, The Centre for Biomedical Engeenering, Zakopiańska 73, 30-418 Krakow, Poland; 4 Institute for Medical Informatics, University of Lübeck, Ratzeburger Allee 160, 23562 Lübeck, Germany; 5 Department of Clinical Engineering, Academy of Silesia, Rolna 43, 40-555 Katowice, Poland; 6 Faculty of Applied Mathematics, Silesian University of Technology, Kaszubska 23, 44-100 Gliwice, Poland; mh308176@student.polsl.pl; 7 German Research Center for Artificial Intelligence, Ratzeburger Allee 160, 23562 Lübeck, Germany

**Keywords:** gesture recognition, smart glove, wearable devices, dynamic gesture

## Abstract

In the evolving field of human–computer interaction (HCI), gesture recognition has emerged as a critical focus, with smart gloves equipped with sensors playing one of the most important roles. Despite the significance of dynamic gesture recognition, most research on data gloves has concentrated on static gestures, with only a small percentage addressing dynamic gestures or both. This study explores the development of a low-cost smart glove prototype designed to capture and classify dynamic hand gestures for game control and presents a prototype of data gloves equipped with five flex sensors, five force sensors, and one inertial measurement unit (IMU) sensor. To classify dynamic gestures, we developed a neural network-based classifier, utilizing a convolutional neural network (CNN) with three two-dimensional convolutional layers and rectified linear unit (ReLU) activation where its accuracy was 90%. The developed glove effectively captures dynamic gestures for game control, achieving high classification accuracy, precision, and recall, as evidenced by the confusion matrix and training metrics. Despite limitations in the number of gestures and participants, the solution offers a cost-effective and accurate approach to gesture recognition, with potential applications in VR/AR environments.

## 1. Introduction

In the current dynamic technological landscape, the advancement of contemporary user interfaces is becoming a crucial focus of research and design. Some prominent examples of such research projects involve the development of interactive systems that improve interpersonal communication and human–computer interaction (HCI) [1] through gestures. In the realm of human–machine interaction, gesture recognition is seen as the most intuitive and natural approach. Consequently, its development is continually advancing, driven by enhancements in the sensors that capture gestures. The technology using gloves for non-verbal communication has been continuously developed since the 1980s [2].

Gestures are identified using deflection sensors, gyroscopes, and video camera images. There are also electromagnetic systems that can locate an object’s position by measuring the electromagnetic fields generated by a transmitter; for example, radio frequency [3]. In the literature, there are mainly two approaches to gesture recognition based on instrumented sensor technology and computer vision [4].

The vision-based approach involves processing digital images and videos using machine learning and deep learning techniques for gesture recognition [5,6]. Although cameras are inexpensive, the main disadvantage of this approach is the complex and time-consuming data processing required to recognize hand gestures, which is hindered by background noise, distance range, multi-gesture problems, varying lighting conditions, the effect of occlusions, processing time traded against resolution and frame rate [7,8,9]. The foreground or background objects that present the same skin color tone or otherwise appear as hands are also problematic.

The second approach gesture recognition relies on sensors most often worn on or embedded in gloves. Sensor-based gesture recognition uses them to detect and measure bending angles, movements, orientations, and alignments of the fingers, as well as the positioning of the palm. These measurements are then used to identify and interpret gestures. Changes in hand gestures are the result of muscle contractions and tendon shifts in the arm and wrist, along with deformations in blood vessels and bone movements. When the hand and fingers move, various biological and physical characteristics are altered. These alterations can be detected using electrical, mechanical, acoustic/vibrational, or optical sensing techniques [10]. The following sensors can be used in gesture recognition systems [10,11]:

- Electrical Sensing: surface electromyography (sEMG) [12], EIT [13];
- Mechanical Sensing: forcemyography (FMG), IMU, strain sensing, flex sensor sensing;
- Acoustical/Vibratory Sensing: sonomyography (SMG), mechanomyography (MMG), bone-conducted sound sensing;
- Optical Sensing: photoplethysmography (PPG).

Relying on a single sensor or multiple same sensors is less desirable as it suffers from several issues limited spatial coverage, limited precision, and uncertainty [11] although we can find many works on data gloves based on the same type of sensor [6,14,15,16,17]. Wearable resistive sensors can only measure the bending angle during joint movement and are unable to determine the spatial orientation of joints due to insufficient information about their spatial distribution [18].

IMU sensors face a well-known problem in which rotation angles are determined by integrating inertial signals, causing errors to accumulate over time [19,20]. Another disadvantage of IMU sensors is the complexity of the calculation process to locate an object [21,22]. One way to overcome these limitations is by employing multisensory fusion to create robust sensing systems using multiple sensors. Sensor fusion combines various sensing modalities with data-fusion techniques to compensate for the shortcomings of individual modalities, providing the hand gesture recognition (HGR) algorithm with comprehensive information to accurately associate gesture or movement patterns [11]. Research indicates that multisensory modalities can interpret hand movements with greater accuracy than unimodal signals. The combination of multiple sensing modalities is an effective solution, allowing them to compensate for the limitations of each other [11,23,24,25,26]. Gesture recognition can be classified according to temporal relationships into two types of static and dynamic forms [27].

- Static Gesture Recognition: Involves gestures where the hand position remains constant during the gesture period and focuses on the shape and flexion angles of the hand. A static gesture is one where the movement of the hand is not the focus; instead, the emphasis is on a specific hand configuration and pose, captured in a single image [28,29]. A signal value is obtained from each sensor, independently of time.
- Dynamic Gesture Recognition: Pertains to gestures where the hand position changes continuously during the gesture period. It involves tracking hand trajectories and orientations over time. Considering not only the shape and flexion angles of the hand, but also the movement patterns [27,30,31]. Dynamic gestures contain additional temporal information, such as ulnar rotation or changes in finger poses (e.g., spreading previously closed fingers) [32,33]. They generally have three motion phases: preparation, stroke, and retraction [34]. The dynamic gesture uses movement and shape as the key point of the gesture [29]. The data collected by the sensors undergo temporal changes and are systematically recorded and analyzed.

Most publications on data gloves describe static gesture recognition [31,33]. As Pan et al. noted in [20], 51.11% of the studies from 2015 to 2022 focused on static gestures, while only 31.11% addressed dynamic gestures. The remaining 17.78% of the significant studies included both static and dynamic gestures.

On the global stage, there are nearly two hundred different sign languages. Some of them enjoy popularity among a wide range of users, reaching hundreds of thousands of people, as is the case with American Sign Language (ASL), commonly used in the United States and Canada. Other sign languages are limited to small rural communities, as is often the case with many local sign languages in Africa and Asia. It should be noted that deaf people from one country usually use the same sign language. Nevertheless, there are significant differences between the sign language and the spoken language used in a given area, suggesting that sign languages do not necessarily derive directly from the spoken languages used in the region [35].

Communication in sign language plays a crucial role in interactions between deaf people and within deaf communities. The central element of this system is the ideographic signs, which often correspond to single words or short phrases, such as idioms. They are accompanied by dactylographic signs, including letters of the alphabet, numbers, punctuation, and mathematical symbols. These signs allow for the transmission of more detailed information, such as proper names, specialized terms, or foreign words. Performing a sign requires knowledge of its sublexical elements, which are described through the following:

- The arrangement of fingers on the hand (one or both, depending on whether the sign is one handed or two handed);
- The placement of the hand relative to the body;
- The position of the hand in space;
- The direction of movement;
- Facial expression [36].

### 1.1. Statistics Related to Sign Language Use

According to statistics, about 0.1% of people are deaf-mute, with approximately 0.17% using sign language. As the literature states, American Sign Language is the third most commonly taught language in American higher education [37], with just over 107,000 people learning ASL in post-secondary institutions in 2016. Currently, there are no reliable contemporary statistics on the total number of ASL users [38].

### 1.2. Smart Glove Signal Processing in Selected Expressions of ASL

Smart gloves, equipped with various sensors, have emerged as cutting-edge technology for the recognition and processing of sign language expressions. These gloves capture the sublexical elements of signs, translating physical movements and positions into digital data. This technology holds great promise for improving accessibility to communication for deaf individuals by providing real-time translation of ASL into spoken or written language.

Smart gloves can accurately detect the following:

- The arrangement of fingers on the hand through flex sensors;
- The placement of the hand relative to the body using position sensors;
- The position of the hand in space with the help of accelerometers and gyroscopes;
- The direction of movement through motion sensors;
- Facial expressions by integrating additional facial recognition technologies.

These features enable the precise capture and interpretation of both ideographic and dactylographic signs in ASL. Advanced signal processing algorithms then analyze these data to identify and translate the signs, facilitating effective communication in different languages and contexts.

By integrating smart glove technology with robust signal-processing techniques, it is possible to bridge the communication gap between the deaf community and the public, making ASL more accessible to those who do not know the sign language and supporting the inclusion of deaf individuals in various social, educational, and professional settings.

The dynamic gesture-based control environment is taking interaction to the next level with reverse brain training that can restore lost behavioral, cognitive, and communication skills. This evolution highlights the importance of developing sophisticated data collection systems, such as smart gloves, tailored to these purposes. Therefore, it is necessary to have the latest reviews of related work that analyze and organize knowledge about research in the following areas that mimic human-hand substitutes.

- Human–machine interfaces (HMI) that meet the growing demands for intuitive and effective manipulation.
- Real-time hand gesture recognition using surface electromyography and machine learning that can help improve human–computer interaction.
- Sign language gesture recognition, which serves as a key input method in human–computer interaction (HCI).
- Sensor substitution using artificial receptors connected to the brain via an HMI that could compensate for sensor loss and potentially expand human capabilities beyond current limitations.

### 1.3. Objective and Paper Structure

This study focuses on developing a smart glove equipped with various sensors to accurately capture hand gestures and the HCI and, in this paper, we explore the possibilities of generating precise control signals based on specific sign language or more precisely game control gestures. The work aimed to create a prototype of a glove using cheap and generally commercially available sensors and used neural networks for the classification of dynamic gestures used to control the game.

This paper is organized as follows: The Introduction highlights the background of this study: technical progress in human–computer interaction, gesture recognition, and processing of sign languages. Section 2 discusses the review of the literature on technological advancements in human–computer interaction, focusing on gesture recognition through smart gloves equipped with various sensors. In addition, it covers the use of advanced sensors and machine learning in real-time hand gesture recognition, as well as applications in sensory substitution technologies for the visually impaired and in motor rehabilitation.

Section 3 presents the construction and assembly of the smart glove, including its sensor configuration and data-acquisition process. It also discusses the creation of a gesture data database and the design of a neural network to classify hand gestures based on the collected data. Section 4 evaluates the performance of the gesture recognition classifier using metrics such as accuracy, precision, F1-score, and recall and examines the model’s loss throughout training. It shows the assessment of the classifier performance in predicting hand gestures and the effectiveness of the learning process. Finally, Section 5 summarizes the findings, presents the limitations, and introduces recommendations for future studies.

## 2. Related Work

### 2.1. Human–Machine Interfaces (HMI)

In the field of gesture recognition, sensing touch and force, the delicate human skin and its advanced nervous system, especially the hand, perfectly sense pressure, tension, and bending stimuli. To mimic this ability, flexible touch and force sensors have been developed in various forms, including electronic skin, electronic fabric, and smart contact lenses. These flexible sensors, unlike conventional rigid devices, adapt to curved and soft surfaces, making them ideal for wearable electronics. They offer higher sensitivity and faster response times, often exceeding the performance of human skin. The applications of these sensors are wide ranging, from health monitoring and object recognition to intelligent robots and human–machine interaction (HMI).

This section reviews significant developments and research in the field of flexible touch and force sensors, focusing on their application in HMI. It highlights a variety of sensor types, such as resistive, capacitive, piezoelectric, and triboelectric sensors, each of which has unique properties and suitability for different HMI applications. The review also explores innovative strategies to improve sensor performance, such as improving sensing range, sensitivity, and multidimensional touch sensing. Furthermore, it investigates the integration of these sensors with HMIs for advanced applications such as robot control and VR/AR technology, demonstrating the transformative potential of these novel HMIs [39].

Zhu et al. developed a touch-sensitive glove equipped with triboelectric sensors and piezoelectric stimulators, designed specifically for virtual-space interaction. Their research addresses the increasing demands for intuitive and effective manipulation within human–machine interfaces (HMIs). The smart glove they propose features triboelectric finger-bending sensors, a hand displacement sensor, and piezoelectric mechanical stimulators, enabling the detection of omnidirectional bending and sliding events in virtual environments. Furthermore, the glove uses machine learning to achieve an object recognition accuracy of up to 96%, showcasing its potential for low-cost advanced HMI applications in various fields such as entertainment, healthcare, sports training, and the medical industry [40].

He et al. developed a glove-based human–machine interface (HMI) utilizing triboelectric nanogenerators (TENGs) for diverse applications. The minimalist design features PEDOT-coated textile strips and silicone rubber thin film to balance full functionality with simplified signal processing. This glove-based interface has been successfully used for wireless car and drone control, VR game control, and cursor control for online shopping. Their innovative approach offers a flexible and user-friendly HMI solution, different from traditional rigid and bulky interfaces [14,41].

Luo et al. developed a glove-based multidimensional human machine interface (HMI) utilizing a bending-angle triboelectric nanogenerator (BA-TENG) for high-resolution finger motion sensing. The system, enhanced by a custom PCB, shows high sensitivity and low crosstalk, improving the signal-to-noise ratio by 19.36 dB. Their HMI effectively supports applications in smart home control, advanced robotics, and a virtual keyboard with user recognition, achieving a classification accuracy of 93.1%. This BA-TENG-based smart glove offers a minimalist and intuitive solution for diverse fields, including IoT, assistive technology, and intelligent recognition systems [42].

### 2.2. Real-Time Hand Gesture Recognition

In the realm of improving human–computer interaction (HCI) through real-time hand gesture recognition (HGR), recent studies have made significant strides. Jaramillo-Yánez conducted a systematic review of the literature focused on state-of-the-art HGR models that use surface electromyography (EMG) data and machine learning techniques [43]. This comprehensive review assessed 65 primary studies, applying Kitchenham’s methodology to analyze data acquisition, segmentation, preprocessing, feature extraction, classification, postprocessing, real-time processing, gesture types, and evaluation metrics. The findings underscored advances in HGR methodologies, emphasizing their role in fostering intuitive and efficient communication within HCI systems.

Fang’s research introduces an innovative data glove that incorporates inertial and magnetic measurement units (IMMUs) to facilitate comprehensive gesture capture and recognition in human–robot interaction (HRI) scenarios [44]. This glove integrates 18 compact and cost-effective IMMU modules, including gyroscopes, accelerometers, and magnetometers, allowing precise tracking of three-dimensional movements of arms, hands, and fingers. Experimental validation highlighted the efficacy of extreme learning machine (ELM) algorithms for static and dynamic gesture recognition, underscoring the potential of IMMU-based systems to enhance gesture-based interaction paradigms.

Dong’s study presents a novel approach to gesture recognition in HMI using a low-cost data glove with a simplified hardware design [45]. Their research focuses on capturing simultaneous finger movement and bending with high accuracy. The proposed dynamic hand gesture recognition algorithm (DGDL-GR) integrates a fusion model of convolutional neural networks (fCNNs) and temporal convolutional networks (TCNs). This model extracts time-domain and spatial-domain features using causal and dilation convolutions to effectively handle sequence-modeling tasks. Experimental results validate the superior performance of the DGDL-GR algorithm in accuracy, F1 score, precision score, and recall score with real-world datasets, highlighting its potential for advanced gesture recognition applications.

Mummadi’s investigation centers on augmenting HCI through data gloves, addressing challenges where external sensors may inadequately capture hand movements [46]. Their proposed data glove integrates an embedded gesture classifier using inertial measurement units (IMUs) on the fingertips, achieving a mean precision of 92% and an F1 score of 91% on 22 gestures from French Sign Language (LSF) in extensive participant trials. Comparative analysis with local fusion algorithms demonstrated improved settle times and reduced delays after gesture changes, facilitating real-time gesture recognition within 63 milliseconds for seamless interaction via Bluetooth-connected systems.

Naser et al., in study [47], present a multi-layer neural network with an autoencoder that recognizes five hand gestures (fist, open hand, wave in, wave out, and double tap) from sEMG signals recorded with a Myo armband with an accuracy of 99.68%, 100%, and 99.26% during training, validation, and testing, respectively. Their proposed multi-layer neural network outperformed the K-nearest neighbor classifier that served as a reference (accuracy of 97%).

### 2.3. Sign Language Gesture Recognition

A specific area of language gesture recognition involves the development of sensory gloves for state-of-the-art sign language recognition between 2007 and 2017 [48]. Gałka’s research contributes to advancing automatic sign language recognition beyond vision-based systems, which are sensitive to environmental changes. Gałka introduces an accelerometer glove designed for robust gesture recognition in sign language [49]. The glove integrates inertial motion sensors and a specialized gesture-acquisition system. Evaluation using Hidden Markov Models (HMMs) and parallel HMM approaches demonstrates a significant reduction in the equal error rate, while maintaining a high recognition accuracy of 99.75%. This approach offers a promising solution to improve the reliability and usability of sign language recognition systems in various recording conditions.

Bhaskaran et al. propose a smart glove capable of converting sign language gestures into speech output, addressing communication challenges faced by people with speech impairments [50]. The glove utilizes flex sensors and an Inertial Measurement Unit (IMU) for gesture recognition, along with a novel State Estimation method to track hand motion in a three-dimensional space. Tested with Indian Sign Language, the prototype demonstrates feasibility in real-time sign language to voice conversion, with potential applications in gaming, robotics, and healthcare.

Similarly, Sa et al. explored the domain of Sign Language Recognition and highlighted the diversity of solutions available for translating hand gestures into text and/or audio output [51]. Their work focuses on improving the accessibility of MEMS accelerometers and the cost-effectiveness of gesture recognition gloves, reducing overall costs compared to traditional flexible sensor-based solutions. This approach aims to make the recognition technology of sign languages more affordable and accessible to almost all human languages, or even more, such as “third hand” [52,53].

Hands play a crucial role in basic daily tasks, and impairments due to neurological conditions can significantly affect one’s quality of life. Wearable hand gesture interfaces promise to restore and aid hand function, while also enhancing communication between individuals and with computers. This review of related works summarizes recent advancements in sensing interfaces and algorithms for hand gesture recognition, applicable across diverse fields such as rehabilitation, prosthesis control, exoskeleton development, sign language interpretation, human–computer interaction, and user authentication. Current findings underscore electrical, mechanical, acoustical, vibrational, and optical sensing as primary input modalities for gesture recognition, with algorithms ranging from classification of fixed hand poses to regression of continuous finger and wrist joint angles. Both conventional machine learning techniques and more recent deep learning approaches have been pivotal in improving the accuracy and versatility of gesture recognition systems, paving the way for future research to focus on improving dataset sizes, ensuring robustness for everyday use, and refining user interface designs to be less obtrusive [10].

### 2.4. Sensory Substitution beyond Current Limitations

Kilian et al. implemented and evaluated the Unfolding Space Glove, an open-source sensory substitution device that transmits the relative position and distance of nearby objects as vibratory stimuli to the back of the hand. This technology enables blind individuals to explore their surroundings in a natural way, aiding in tasks such as object recognition and navigation [54].

Mendes et al. investigated cortical audiotactile integration mechanisms using a sensor glove, aiming to preserve the cortical map of the hand after peripheral nerve injuries. Their findings suggest that sensory substitution through auditory-tactile interfaces can establish connections between auditory and somatosensory cortical areas, influencing neural plasticity and enhancing sensory perception [55].

Paterson et al. discussed the historical context and development of sensory substitution systems, highlighting early experiments such as Project Felix and tactile-visual substitution systems pioneered by Paul Bach-y-Rita. Their work underscores the foundational role of neuroplasticity in the evolution of sensory substitution technologies [56].

Chen et al. presented a wearable hand rehabilitation system that integrates a sensory glove with flex sensors for motion detection and motor assistance, facilitating mirror therapy and task-oriented training for stroke patients. This system demonstrates high accuracy in gesture recognition and supports functional grasp rehabilitation through sensorimotor feedback [57].

Kim et al. developed an e-glove system for prosthetic hands, combining stretchable sensors and soft actuators to replicate human hand-like sensory perceptions. This system improves user comfort and interaction capabilities, addressing challenges in sensory integration for amputees [58].

Hafidh et al. introduced the F-Glove, a sensory substitution system aimed at enhancing grip force modulation in diabetic patients using pressure sensors. This system provides auditory feedback proportional to the pressure of the fingertip, which helps manipulate objects and restore sensory function [59].

Demolder et al. reviewed recent advances in wearable sensing gloves and sensory feedback devices, emphasizing their applications in healthcare, prosthetics, and virtual reality. They discussed the integration of soft actuators and bioelectronics in developing lightweight and ergonomic devices that enhance sensory perception and rehabilitation of motor function [60].

Liu et al. investigated the use of an instrumented glove to enhance motor learning through sensory feedback and agency perception. Their findings suggest that real-time feedback improves grasp performance and cognitive engagement during rehabilitation, showcasing the potential for sensory substitution for functional recovery in clinical settings [61].

Table 1 summarizes data from 10 sample articles focused on the development and research of data gloves, published within the last five years, with most of them appearing in the past two years. As shown by the data in Table 1, the majority of data gloves were used to analyze static gestures, and the quality of the classifier was determined based on the accuracy metric. The use of flex sensors in these types of solutions remains very popular. IMU sensors are increasingly being used in data gloves, replacing the separately used gyroscopes and accelerometers. Utilizing more than one IMU sensor can improve the efficiency of the classifier. Scientists are continually searching for new tools to recognize and classify different types of gestures.

## 3. Materials and Methods

**Figure 1:**
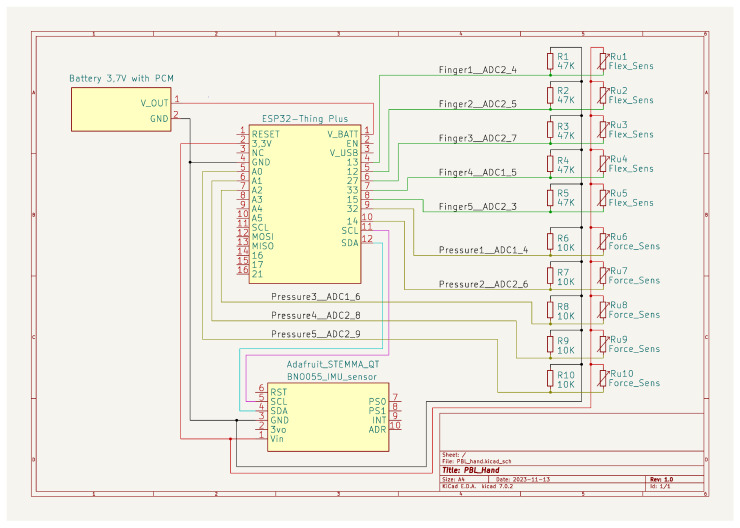
Electrical schematic of constructed glove.

To maintain the integrity of the process, an electrical diagram of the device was drawn before assembly, including all signals and power connections. The glove schematic, shown in Figure 1, was prepared in KiCad version 8 software publicly available under the GNU General Public License version 3 from [68]. The entire device is based on the SparkFun ESP32-S2 microcontroller. This microcontroller offered the peripherals required for the project as well as plenty of computing power to allow further development of the project and implementation of simple machine learning algorithms for real-time classification of read-out movements.

A power supply was provided by a battery connected to the microcontroller or an external source connected to the USB-C port. All of the sensors used can operate at 3.3 V, so no additional inverter was required to convert the power to 5 V, and the microcontroller’s available power supply was used. In the diagram, to the right of the microcontroller, there are two sets of sensors—deflection and pressure—in a group of five, one for each finger of the hand. As the flexion and pressure sensors work by changing resistance when they are bent or pressed, and the microcontroller is only able to measure the voltage connected to the analog pin, using an internal 13-bit ADC, it was necessary to convert this to voltage changes before connecting the signal to the analog pins.

For this purpose, a simple voltage divider was assembled around each sensor. A supply voltage was connected to one sensor lead. The other lead was connected to the subsequent analog pins assigned to each sensor. In parallel, this signal through a sensor-appropriate resistor (for the deflection sensors it was a 47 kΩ resistor, and for the pressure sensors a 10 kΩ resistor) was connected to the ground, with the result that voltage changes corresponding to changes in sensor resistance were observed on the analog pin. The value of the resistors was chosen to correspond to the resistance obtained at half the measured range. Thus, half of the supply voltage determined this point. As each sensor had a different resistance at rest and during assembly there were no sufficiently precise resistors with resistances corresponding to the sensors’ resistances at rest, the same resistor was used for each sensor, with low accuracy (1%).

This resulted in a different measurement range for each sensor. To overcome these problems, the change in value during finger movement was analyzed, rather than the value itself in a specific state; in addition, appropriate thresholds were adopted for unambiguous gestures, which determine whether the read-out value corresponds to a bent or upright sensor. Another important module, located on the schematic below the microcontroller, is the 9-axis IMU sensor, communicating via the I^2^C bus. Thanks to the on-chip software, linear velocity and angular position information, determined from the position of the accelerometer and gyroscope, can be read directly from the module. These data are important in the glove’s role as a precision controller, as it allows hand movements to be mapped in a 3D computer environment such as Unity.

A standard construction glove was used as the base for assembling the components. In the first stage of assembly, the method of mounting the deflection sensors was tested. It was decided to mount the sensor on the end with the base hanging freely while working when the finger is bent. The sensor on the glove was glued with silicone adhesive. It was placed slightly above the line of inflection marking the upper phalanx of the finger. In the space left, at the tip of the finger, the pressure sensor was glued using the same adhesive. Before gluing itself, the surface of the sensor was scratched with a sharp tool to increase adhesion. The assembly was repeated for all five fingers. In the next step, a voltage divider circuit was assembled around the leads of each of the ten sensors. Signal wires of appropriate length were soldered to the signal outputs.

The power supplies and grounds were wired together and gathered into a single point, from which individual wires were routed to the corresponding pins on the microcontroller board. The power supplies and ground necessary to create the voltage dividers were grouped in the palm of the hand, in front of the IMU sensor, to minimize the number and length of the signal wires. In the central part of the outside of the palm, the circuit with the BNO055 IMU sensor was attached to the glove using thread. Whenever possible, longer pieces of bare wires were protected with shrink sleeves. The signal connections to the microcontroller were made using AWG28 ribbon strands properly split and cut to the required size. As all wires change position during hand movement, to prevent mechanical damage, each connection has been suitably elongated, and excess wires have been attached to the glove, so that they can work and do not interfere with the use of the glove or become entangled.

To increase the freedom of movement, the large microcontroller board with the battery was placed on the user’s forearm. To this end, a sports tie was used, to which the microcontroller board was attached by thread through the mounting holes. The battery pack was left in a plug-in form so that it could be quickly replaced, measurements carried out with the glove could continue, and in the event of battery degradation to be simply replaced with a new one, terminated with an identical two-pin JST female plug. A zip pocket, located on the wristband, was used to store the battery pack.

### 3.1. Data Acquisition

**Figure 2:**
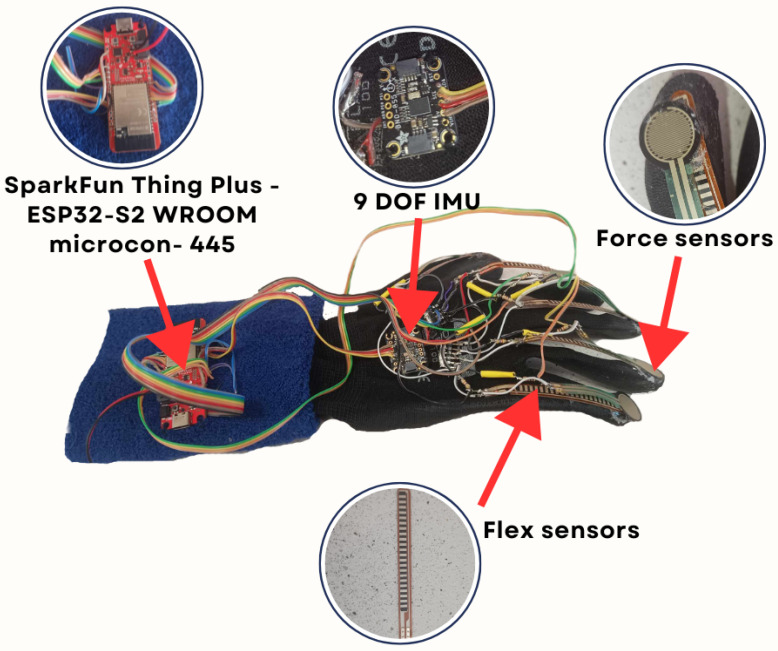
Gesture recognition glove with sensor placement.

The data collection process starts with the configuration of the sensors and microcontroller. Various types of sensors were used, such as a deflection sensor, a force sensor, and an inertial sensor. The SparkFun Thing Plus - ESP32-S2 WROOM microcontroller was used to collect and transmit data from these sensors. The elements are presented in  Figure 2.

The procedure involves calibrating the system, in which the reading parameters are adjusted to match the ranges of motion and pressure force characteristic of different gestures. The system was configured by mounting the sensors onto a test glove and connecting them to the microcontroller. The connections were then tested, and the microcontroller was programmed using MicroPython to collect data.

**Figure 3:**
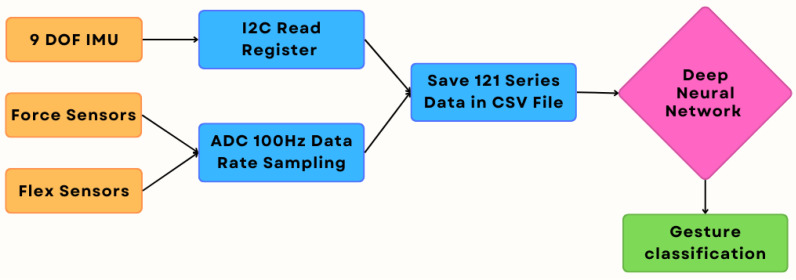
Flowchart of the data acquisition and processing.

The analog signals from the deflection and pressure sensors were converted to digital form in the microcontroller using an ADC. The signals from the inertial measurement units (IMUs) were read out in digital form from the sensor measurement queue via the I^2^C bus interface. To eliminate microvariations, a threshold was introduced for the IMU data, the crossing of which was recorded as a change in value. This eliminated the noise read by the sensors at rest (sensor on the table). Signals are recorded by sending the appropriate command through the USB port. The data are written as CSV files with the given name in the command to start writing to the microcontroller’s flash memory. Gesture recording lasts for a duration of 1210 µs and the data are recorded at a frequency of 100 Hz. Each recording session produced one CSV file. A data flow diagram in Figure 3 has been prepared to better illustrate the signal acquisition, data processing, and classification process.

### 3.2. Virtual Reality Interface

Accurate recognition of hand movements should be integrated into applications for game control. These applications may be developed, for instance, with the Unity 3D engine. The operation of such an application would proceed as follows:

1. Initiation of registration (establishing starting position);
2. Execution of gesture by the user;
3. Termination of signal registration (return to starting position);
4. Signal recognition;
5. Visualization of gesture within the user interface;
6. Execution of the related command.

### 3.3. Experiment Setup

The tests were conducted according to a prepared test procedure. Participants were instructed to perform selected gestures, which were then measured. The procedure involved performing each gesture in 1210 microseconds, during which sensor data were recorded. After each measurement was completed, the data were transferred to a computer for further analysis.

### 3.4. Experimental Group

**Figure 4:**
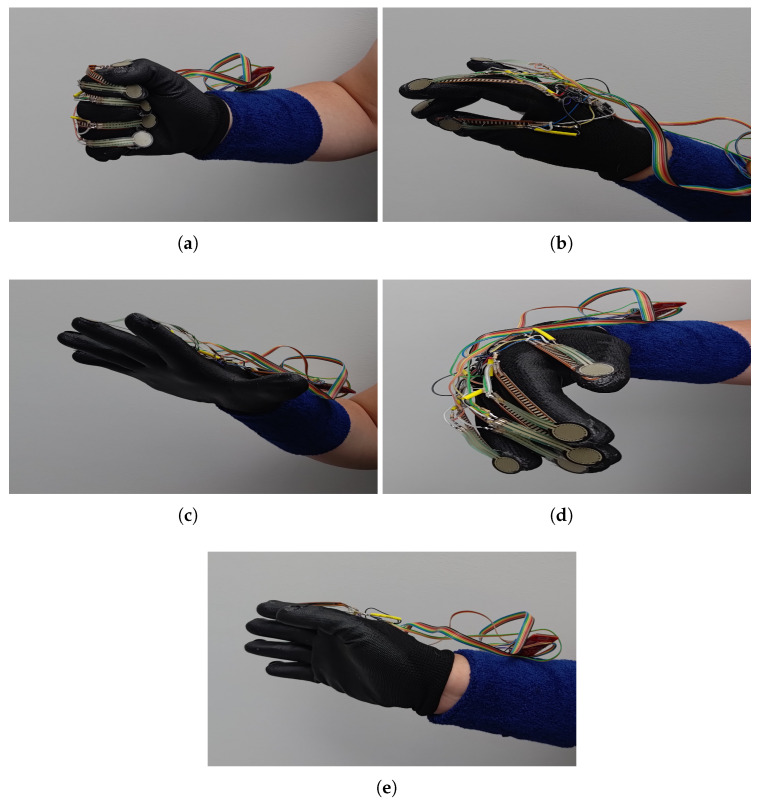
Game control gestures: (**a**) fist; (**b**) double tap; (**c**) finger spread; (**d**) wave left; (**e**)—wave right.

Ten people aged 22 to 59 years participated in the experiment. Eight of them were around 25 years old. There were six men and four women among the participants in the experiment. None of the participants had been diagnosed with neurological diseases. Before performing a series of gestures, the person supervising the experiment trained them on how to perform the gestures. Each phase of the experiment, comprising successive gestures, was carried out under equivalent conditions that guaranteed the subject’s full concentration. Each participant performed two repetitions of each gesture. The gestures performed were used in the MYO game controller and are described in detail by Rawat et al. and Naser and Hashim [47,69]. The following gestures were performed:

1. Fist (Figure 4a);
2. Double tap (Figure 4b);
3. Finger spread (Figure 4c);
4. Wave left (Figure 4d);
5. Wave right (Figure 4e).

Figure 4 shows the gestures performed during the experiment.

### 3.5. The Cost of Producing a Data Glove Prototype

The prototype of the glove was created using five flex sensors (Adufruit Short flex Sensor), five force sensors (SparkFun Force Sensitive Resi sensor), IMU sensors (Adafruit Adafruit 9-DOF), a microcontroller (SparkFun Thing Plus—ESP32-S2 WROOM), a battery, wires, gloves, and a description with a battery pocket. The total cost of materials for glove prototype was estimated at PLN 545 (USD 137). The cost of materials to create the glove was estimated based on the prices offered by retail distributors of electronic components operating on the Polish market. Sensors and other components necessary for the implementation of the project were bought from various distributors in individual quantities, ranging from one to approximately a dozen units.

### 3.6. Expressions of Gestures

We adopted different types of sensors for gesture detection, flex sensors, force sensors, and inertial sensors (IMUs). Flex and force sensors provide the highest precision in detecting finger position, while IMU sensors are useful for monitoring movement and changing hand position in specific axes. The use of various types of sensor prevents the issues described in the Introduction, such as measuring only the finger-bending angle (flexion sensors) or the accumulation of errors over time due to the integration of inertial signals (IMU sensor). The inclusion of force sensors allowed for the determination of whether the fingertips were touching and with what force. The use of the glove itself, along with a relatively large number of different sensors, also has its disadvantages, such as limiting finger mobility and touch sensitivity. The wearing of a glove alone increases the risk of the hands sweating and allergic reactions to the materials used. Another drawback of the presented glove prototype is the possibility of detachment of the flexion or force sensors, as they were attached in only two places to avoid restricting hand mobility or adding extra weight to the hand.

### 3.7. Database

The created data storage of 100 files was not particularly comfortable in analysis and usage. Therefore, it has been merged into one large CSV file, while adding information about patients, which adds up to 27 columns, where six of them are basically metadata and the rest are data stored in lists. Columns in the final CSV file are listed and described below (column names are in **bold**):

- **ID_person**
  A unique number assigned alphabetically to a person’s name.
- **age**
  An integer in the range of uint8 (between 0 and 255) that represents the age of a person at the time of data collection.
- **gender**
  - F—Female
  - M—Male
- **gesture**
  A categorical variable denoting a given gesture (*5 unique gestures*) —those names have been presented in Section 3.4.
- **repetition**
  Number of repetitions of gesture per recording (*always 1*).
- **recording**
  Number of recording gestures (*1* or *2*).
- **time**
  Number of milliseconds from the beginning of the recording.
- **index.bend**, **thumb.bend**, **little.bend**, **middle.bend**, **ring.bend**
  Values from bend resistive sensors placed on *index*, *thumb*, *little*, *middle*, and *ring* fingers, respectively.
- **index.pressure**, **thumb.pressure**, **little.pressure**, **middle.pressure**, **ring.pressure**
  Values from pressure resistive sensors placed on *index*, *thumb*, *little*, *middle*, and *ring* fingers, respectively.
- **imu.orientEulX**, **imu.orientEulY**, **imu.orientEulZ**
  Values from *Euler angles* from the *IMU* sensor placed on the glove.
- **imu.orientQuatX**, **imu.orientQuatY**, **imu.orientQuatZ**, **imu.orientQuatW**
  Values from *Quaternion angles* from the *IMU* sensor placed on the glove.
- **imu.linacceleX**, **imu.linacceleY**, **imu.linacceleZ**
  Values from *linear acceleration* from the *IMU* sensor placed on the glove.

### 3.8. Deep Neural Network Design

Various neural network configurations were experimented with during the project work. On pair with custom-made layers stack, with VGG16 and ResNet50 (customized input and output layers to match desired usage). The final neural network had the following configuration:

- Number of fully connected layers: 2;
- First layer size: 32 filters (Conv2D), 3 × 3 kernel size;
- Second layer size: 64 filters (Conv2D), 3 × 3 kernel size;
- Third layer size: 64 filters (Conv2D), 3 × 3 kernel size;
- Activation function: ReLU (Rectified Linear Unit);
- Iteration limit: 50 epochs;
- Validation frequency: every epoch.

**Figure 5:**
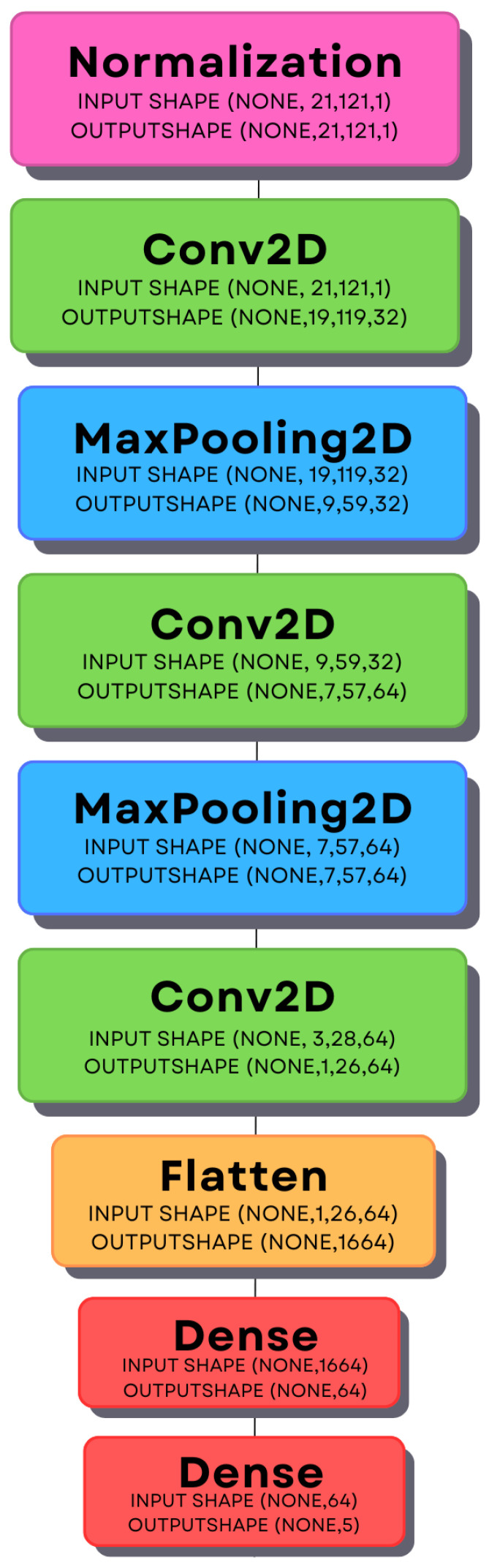
Structure of the deep neural network.

These hyperparameters were determined by iterative testing to achieve the best classification performance and to minimize computational complexity. The detailed model architecture is presented in  Figure 5. That CNN (Convolutional Neural Network) has been used in pair with 5-fold cross-validation. The target outcome for classification was to minimize the value of the loss function in the validation sets. The learning process has been performed on various available personal machines for people who created a dedicated CNN solution for this project, it did not use GPU acceleration, due to inconsistency in used machines, the process was not very demanding, and it took a long time to consider a unified, centralized platform.

The classifier was implemented in Python 3.10 using the Tensorflow 2.16.1 library, as Sequential model, which is a stack of layers, where every layer has exactly one input and output tensor [70]. Each layer behaves differently, according to [71]:

- **Normalization**—preprocessing layer that performs normalization of continuous features, which means that it will shift and scale inputs into a distribution centered around 0 with standard deviation of 1. This is accomplished by pre-computing the mean and variance of the data and calling $\frac{input-mean}{\sqrt{var}}$ at runtime [71].
- **Conv2D**—creates a convolution kernel that is convolved with the input over a dedicated axis to produce a tensor of outputs [71].
- **MaxPooling2D**—downsamples input matrix along height and width (x and y) by taking a maximum value from a window of declared size. This is conducted it for each input channel (3rd dimension) [71].
- **Flatten**—flattens the input, which means squashing one of the dimensions [71].
- **Dense**—implements the operation: output=activation(dot(input,kernel)+bias) where activation is the element-wise activation function passed as the activation argument, kernel is a weight matrix created by the layer, and bias is a bias vector created by the layer (only applicable if use_bias is True) [71].

All Conv2D layers and the first appears Dense layer are using **ReLU** (Rectified Linear Unit) activation function. It selects larger numbers between the current input and 0 (used without modifications). The last Dense layer uses the **softmax** activation function, which is mathematically described in Equation (1), where **z** is the input vector.

(1) $$\sigma \left(z_{i}\right)=\frac{e^{z_{i}}}{\sum_{j=1}^{n}e^{z_{j}}};for\;i=1,2,\ldots ,n$$

Classifier has been fed with an untouched dataset, nothing has been cut, and it has not been filtered in any way. The normalization layer allowed only for the rescaling of data from the original range (the way has been described in the normalization layer description) to 0–1.

## 4. Results

In this section, we present the results of our classifier, which has been trained on a dataset of hand gesture data collected using sensors embedded in a glove.

### 4.1. Performance Metrics

Table 2 shows the performance metrics (accuracy, F1 score, loss, precision, recall) of the proposed classifier.

The results based on the test data show the high efficiency of the model in recognizing hand gestures; the accuracy of 90.00% and F1-score of 0.9132 demonstrate the effectiveness of the classifier in the test set and a good balance between precision and completeness of classification. Although the loss value of 0.5337 is not directly interpretable in terms of classification accuracy, the high values of precision (0.9) and completeness (0.9) confirm the high accuracy in recognizing positive observations and true classes.

### 4.2. Confusion Matrix

**Figure 6:**
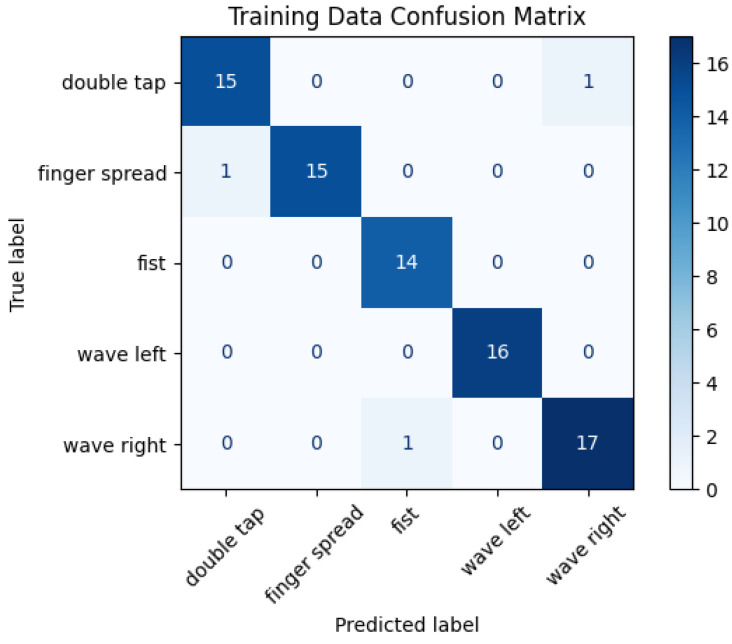
Confusion matrix.

The confusion matrix shown in Figure 6 summarizes the classifier’s predictions on the training data.

The confusion matrix for the training data confirms the high accuracy of the classifier, where most of the predictions were made correctly. Only three cases of confusion were identified, highlighting the stability and effectiveness of the model in identifying different hand gestures at the training level.

### 4.3. Model Loss and Accuracy

**Figure 7:**
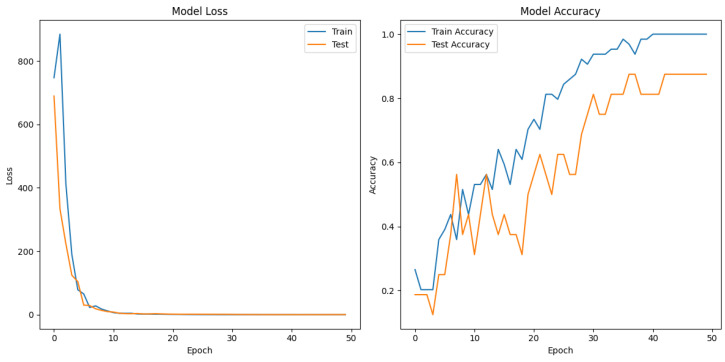
Loss and accuracy of the deep neural network.

In Figure 7, the loss of the model and the accuracy of the model are presented.

In the analysis of experimental results, it was observed that the loss function (*loss*) decreases for both training and test data as the epochs progress, indicating the effectiveness of the learning process. The decrease in loss values reflects the model’s improved fit to the available data and its ability to reduce predictive errors.

Simultaneously, classification accuracy (*accuracy*) increases for both the training and test datasets, demonstrating the model’s capability to accurately identify target classes in new data. The observed increase in accuracy results from the model effectively adapting to various testing conditions and positively evaluating its generalization ability.

## 5. Discussion and Conclusions

We successfully designed and built a glove capable of capturing dynamic gestures designed for game control. The recorded gestures are differentiable enough that it is possible to classify performed gesture accurately with accuracy, precision, and recall of 0.9. The confusion matrix shown in Figure 6 confirms the model’s ability to correctly recognize gestures, with almost non-false positive or negative indications or misclassifications.

The loss and precision of the model during the training epochs shown in Figure 7 present an increasing accuracy and a decreasing loss value, which shows improvements of classification over the training period. Considering a relatively small amount of data, it is possible to conclude that this model is enough for non-crucial, real-world applications within used gestures. These results confirm that the developed model is well suited to the data analyzed and can be used effectively for classification in real-world applications.

The reported recall, precision, and accuracy are similar to those reported in [33], worse than reported in [47,63,65,66,67], and better than reported in [62] (see results in Table 1). The most significant limitation are the number of examined gestures (only five game gestures) and the number of subjects (10) consisting of people without neurological diseases. We did not include testing glove control in real life scenarios due to the lack of access to the application programming interface (API) that could provide integration between gloves and applications. Another limitation is the construction of the smart glove that consists of a microcontroller and sensors attached to a glove with sports ties, knits, and loose wires between them.

The developed solution is an advancement in human–computer interface devices by providing a cost-effective, accurate, and practically applicable form that can be used in various limited applications where approximately five gestures would be enough. Practically, it can be considered as a navigation method in VR/AR environments that could move the main weight from the face of a user to the hands, or limit the currently used motion-tracking cameras on the goggles themselves. This work contributes to the broader field of gesture recognition and wearable technology.

The smart glove designed in this study is not limited to game control. Potential applications may include the control of drones, robots, and other devices. In the future, the system could be improved by extending the number of recognized gestures and could be improved by adding sign language expressions, carrying out tests in a larger and more diverse study group, covering the wires, and optimizing its length, sensors, and microcontroller. We also plan to test the glove in real-life scenarios.

## Figures and Tables

**Table 1:** Summary of studies on model-based classification and sensors for gesture recognition.

Model/Classifier	Performance Metrics ^1^	Gestures/Purpose	Sensors	Reference	Year
Parallel Hidden Markov Model (HMM)	Data from 8 volunteers, recognition accuracy was 85.21%	Korean SLA; data have been collected with 20 DSL words and 14 static gestures to compliment them	WonderBox, WonderSense, 9-axis inertial sensor module, MPU9250, Bend sensor	[62]	2023
Functional test, performed by deaf and mute people	100%Performance based on subjective users opinions	Two-way Sign Language live translation	10 flex sensors	[6]	2023
The voting meta-classifier (VL2)	Independent user testing (DHS): 87.50% for 56, 91.91% for 27 93.28% for 56, 95.55% for 27	Static geastures: 2 different datasets: 27 gestures from ASL, finger alphabet, Sign Language Lexicon (56 hand shapes)	Magnus Prime X data gloves 9 degrees of freedom (DoF) IMUs 2D flex sensor DoF IMU	[33]	2023
STFGes with LE-ConvLSTM and MSFF	Accuracy: 0.970 ± 0.017 Precision: 0.971 ± 0.014 Recall 0.970 ± 0.020F1 score: 0.970 ± 0.018	10 Chinese Sign Language (CSL) expressions: hello, bye, eat, finish, like, who, not, drink, amazed, very	5 strain sensors, 3–axial accelerometer	[26]	2023
Spatio-temporal feature extraction Hidden Markov Models	Average recognition rate of words: 94%	Static: Arabic Sign Language	Flex sensors, contact pad, MPU5060 accelerometer and gyroscope	[63]	2020
Compared to reference WT9011DCL industrial sensor	Static and dynamic error: 0.32°; 1.11°; 2.61°; ±3° thumb; ±2° index	Open hand, half closedRehabiliatation and game control	15 LSM6DS3 IMUs	[33]	2023
Forward dynamic model (FDM)	87% of the natural object width	Dynamic: rhythmic piano playing and car racing; parallel gripper cont.	Piezoresistive tactile sensor; vibrotactile haptics feedback using embroided copper coil	[64]	2024
Attention-based CNN-BiLSTM network	Acc. 95.05% Prec. 95.43% Recall 95.25%F1 score 95.22%	Spatio-temporal features of dynamic gestures	VRTRIXTM Data Glove using 11 sensors (9 DOF IMU)	[65]	2023
A table with specified values for a given character lying within the defined range	94% sign recognition accuracy	Static gestures (26 ALS signs, 15 simple words)	Flex sensor, 1 for thumb and pinky, 2 for rest finger;MPU-6050 accelerometer and gyroscope	[66]	2019
Gated recurrent unit (GRU algorithm)	92%	DG involving movements of different fingersRecognition of a sequence of a finger gesture	Flex sensors	[67]	2024

^1^ Accuracy if no metric was specified.

**Table 2:** Model performance metrics.

Metric	Value
Accuracy	90.00%
F1-score	0.9132
Loss	0.5337
Precision	0.9
Recall	0.9

## Data Availability

The original data presented in this study are contained within the Supplementary Material.

## Supplementary Materials

The following supporting information can be downloaded at: https://www.mdpi.com/article/10.3390/s24186157/s1, README 1. game_gestures.db.
