# Supplementary material for: Machine Learning-Based Gesture Recognition Glove: Design and Implementation
Source: Sensors (Basel). 2024 Sep 23;24(18):6157. doi: 10.3390/s24186157 (PMC11435472; doi:10.3390/s24186157)
Supplement: Supplementary file 1 [file sensors-24-06157-s001.zip › README 1.pdf]

Data model

Dataframe is stored in a *csv* file as defined in the model below:

```
ID_person       :   int
age             :   int
gender          :   'F' | 'M'
gesture         :   'fist' | 'double tap' | 'finger spread' | 'wave left' |
'wave right'
repetition      :   1
recording       :   1 | 2
time            :   List[int(0 - 1200)]
index.bend      :   List[int(0 - 8191)]
thumb.bend      :   List[int(0 - 8191)]
little.bend     :   List[int(0 - 8191)]
middle.bend     :   List[int(0 - 8191)]
ring.bend       :   List[int(0 - 8191)]
index.pressure  :   List[int(0 - 8191)]
thumb.pressure  :   List[int(0 - 8191)]
little.pressure :   List[int(0 - 8191)]
middle.pressure :   List[int(0 - 8191)]
ring.pressure   :   List[int(0 - 8191)]
imu.orientEulX  :   List[int(0 - 8191)]
imu.orientEulY  :   List[int(0 - 8191)]
imu.orientEulZ  :   List[int(0 - 8191)]
imu.orientQuatX :   List[int(0 - 8191)]
imu.orientQuatY :   List[int(0 - 8191)]
imu.orientQuatZ :   List[int(0 - 8191)]
imu.orientQuatW :   List[int(0 - 8191)]
imu.linacceleX  :   List[int(0 - 8191)]
imu.linacceleY  :   List[int(0 - 8191)]
imu.linacceleZ  :   List[int(0 - 8191)]
```

Meaning of each column is described in next subsections

ID_person

unique number assigned alphabetically to person's name

age

integer basically in the range of uint8 that represents person's age in the moment of collecting data

gender

- F - Woman
- M - Man

gesture

A categorical variable denoting a given gesture.
5 unique gestures

repetition

Number of repetition of gesture per recording.
In this case **always 1**

recording

Number of recording gesture.
Here it can be either:

- **1**
- **2**

time

Number of milliseconds from the start of recording

index.bend

Value from bend resistive sensor placed on **index finger**

thumb.bend

Value from bend resistive sensor placed on **thumb finger**

little.bend

Value from bend resistive sensor placed on **little finger**

middle.bend

Value from bend resistive sensor placed on **middle finger**

ring.bend

Value from bend resistive sensor placed on **ring finger**

index.pressure

Value from pressure resistive sensor placed on **index finger**

thumb.pressure

Value from pressure resistive sensor placed on **thumb finger**

little.pressure

Value from pressure resistive sensor placed on **little finger**

middle.pressure

Value from pressure resistive sensor placed on **middle finger**

ring.pressure

Value from pressure resistive sensor placed on **ring finger**

imu.orientEulX

Value from **Euler angle** from **IMU** sensor placed on the glove

imu.orientEulY

Value from **Euler angle** from **IMU** sensor placed on the glove

imu.orientEulZ

Value from **Euler angle** from **IMU** sensor placed on the glove

imu.orientQuatX

Value from **Quaternion angle** from **IMU** sensor placed on the glove

imu.orientQuatY

Value from **Quaternion angle** from **IMU** sensor placed on the glove

imu.orientQuatZ

Value from **Quaternion angle** from **IMU** sensor placed on the glove

imu.orientQuatW

Value from **Quaternion angle** from **IMU** sensor placed on the glove

imu.linacceleX

Value from **linear acceleration** from **IMU** sensor placed on the glove

imu.linacceleY

Value from **linear acceleration** from **IMU** sensor placed on the glove

imu.linacceleZ

Value from **linear acceleration** from **IMU** sensor placed on the glove

Data values

Columns:

- ID_person
- age
- gender
- gesture
- repetition
- recording

always contains single value, string or number, but just a 1 value.

Rest of the columns are `lists` of length ***121***.

The length

Why ***121*** values? Because **time** of recording was `1.21 seconds` with **frequency** of `100 Hz`.
It can be seen in `time` column, where maximum value is **1200** and minimum is **0**, so overall **1210 values**

Values range

Time

- minimal: 0
- maximum: 1200

That is because it just counts `fs` during recording data. It is given in **ms**.

Sensors

Used micro-controller have **13-bit** ADC which allows that values range.
However *real* value range on every sensor differs, because not every time, for example sensors that relay on changing resistance, doesn't always have 0 Ω value in one extreme state and maximum that '*stops*' all voltage within given current.

Data load

To load data into python program, the following command from `pandas` package can be used:

```
from pandas import read_csv, DataFrame

data: DataFrame = read_csv(dir_path, sep=';')
```
